# Inositol pyrophosphates regulate RNA polymerase I-mediated rRNA transcription in *Saccharomyces cerevisiae*

**DOI:** 10.1042/BJ20140798

**Published:** 2015-02-06

**Authors:** Swarna Gowri Thota, C. P. Unnikannan, Sitalakshmi R. Thampatty, R. Manorama, Rashna Bhandari

**Affiliations:** *Laboratory of Cell Signalling, Centre for DNA Fingerprinting and Diagnostics, Nampally, Hyderabad, Telangana, India; †Graduate Studies, Manipal University, Manipal, Karnataka, India

**Keywords:** diphosphoinositol pentakisphosphate, inositol hexakisphosphate kinase, inositol pyrophosphate, Kcs1, pyrophosphorylation, 1PP-IP_5_/1-IP_7_, 1-diphosphoinositol pentakisphosphate, 1,5[PP]_2_-IP_4_/IP_8_ bis-diphosphoinositol pentakisphosphate, 5PP-IP_5_/5-IP_7_, 5-diphosphoinositol pentakisphosphate, 6AU, 6-azauracil, CDK, cyclin-dependent kinase, DEPC, diethyl pyrocarbonate, ETS, external transcribed spacer, IP_6_, inositol hexakisphosphate, IP_7_, diphosphoinositol pentakisphosphate, ITS, internal transcribed spacer, NTP, nucleoside triphosphate, NTS, non-transcribed spacer, Pol I, RNA polymerase I, qPCR, quantitative PCR, SC, synthetic complete, SC−Met, synthetic complete medium without methionine, SC−Ura, synthetic complete medium without uracil, UAF, upstream activating factor, WT, wild type, YPD, yeast extract peptone dextrose

## Abstract

Ribosome biogenesis is an essential cellular process regulated by the metabolic state of a cell. We examined whether inositol pyrophosphates, energy-rich derivatives of inositol that act as metabolic messengers, play a role in ribosome synthesis in the budding yeast, *Saccharomyces cerevisiae*. Yeast strains lacking the inositol hexakisphosphate (IP_6_) kinase Kcs1, which is required for the synthesis of inositol pyrophosphates, display increased sensitivity to translation inhibitors and decreased protein synthesis. These phenotypes are reversed on expression of enzymatically active Kcs1, but not on expression of the inactive form. The *kcs1*Δ yeast cells exhibit reduced levels of ribosome subunits, suggesting that they are defective in ribosome biogenesis. The rate of rRNA synthesis, the first step of ribosome biogenesis, is decreased in *kcs1*Δ yeast strains, suggesting that RNA polymerase I (Pol I) activity may be reduced in these cells. We determined that the Pol I subunits, A190, A43 and A34.5, can accept a β-phosphate moiety from inositol pyrophosphates to undergo serine pyrophosphorylation. Although there is impaired rRNA synthesis in *kcs1*Δ yeast cells, we did not find any defect in recruitment of Pol I on rDNA, but observed that the rate of transcription elongation was compromised. Taken together, our findings highlight inositol pyrophosphates as novel regulators of rRNA transcription.

## Abstract 2

Inositol pyrophosphates are phosphate-rich metabolic messengers that regulate many cellular processes. We observed that RNA polymerase I is pyrophosphorylated by inositol pyrophosphates, and its transcription elongation activity was reduced in budding yeast strains devoid of inositol pyrophosphates.

## INTRODUCTION

The biosynthesis of ribosomes is a complex biological process that starts with the transcription of pre-rRNA by RNA polymerase I (Pol I) in the nucleolus. In the budding yeast *Saccharomyces cerevisiae*, an extensively used model system to study ribosome biogenesis, 35S pre-rRNA is co-transcriptionally processed to yield mature 18S rRNA, which is part of the 40S ribosomal subunit, and 5.8S and 25S rRNA, which together with 5S rRNA are part of the 60S ribosomal subunit [1,2]. These subunits assemble on mRNA in the cytoplasm to form the 80S ribosomal particle responsible for protein synthesis. As ribosome biogenesis accounts for approximately 80% of a cell's energy consumption [3], it needs to be co-ordinated with the need for protein synthesis to avoid wasteful energy expenditure, and is therefore tightly regulated by the metabolic status of a cell.

Inositol pyrophosphates are energy-rich signalling molecules that contain one or more diphosphate or pyrophosphate moieties, and act as metabolic messengers, regulating energy homoeostasis in eukaryotic cells [4–6]. The most abundant inositol pyrophosphate, 5PP-IP_5_ (5-diphosphoinositol pentakisphosphate or 5-IP_7_), is synthesized from inositol hexakisphosphate (IP_6_) in *S. cerevisiae* by the enzyme IP_6_ kinase or Kcs1 [7]. The 5PP-IP_5_ is further phosphorylated by Vip1 to generate 1,5[PP]_2_-IP_4_ (bis-diphosphoinositol pentakisphosphate or IP_8_) [8,9]. Vip1 also synthesizes 1PP-IP_5_ (1-IP_7_) from IP_6_, which in turn is converted into IP_8_ by Kcs1 [5,8,10,11]. The β-phosphate group at both positions 1 and 5 is removed by the enzyme diphosphoinositol polyphosphate phosphohydrolase (Ddp1) in *S. cerevisiae*, to convert IP_8_ and both forms of IP_7_ into IP_6_ [12]. The *kcs1*Δ yeast strains had undetectable levels of IP_7_ and IP_8_ [13,14], and displayed slow growth, temperature sensitivity [13,15], an impaired stress response [13,16] and altered cellular energy dynamics [17]. Other phenotypes resulting from the loss of Kcs1 in yeast cells include defects in DNA repair, vacuole morphology, autophagy, telomere length regulation, cell cycle progression, polyphosphate levels and inositol metabolism [5,6,18]. On the other hand, *vip1*Δ yeast cells have increased levels of 5-IP_7_ [14], display resistance to hydrogen peroxide-induced cell death [14] and have defects in phosphate homoeostasis [19]. As Ddp1 prefers 1-IP_7_ as a substrate over 5-IP_7_ [12,20], the deletion of both Kcs1 and Ddp1 in yeast cells leads to the accumulation of 1-IP_7_ [8]. Inositol pyrophosphates regulate multiple cellular pathways by two molecular mechanisms [6]: (i) by directly binding to proteins and modulating their function, and (ii) by protein pyrophosphorylation, in which a β-phosphate is transferred from inositol pyrophosphates to pre-phosphorylated serine residues surrounded by acidic amino acids. Modulation of protein function by binding is specific to individual inositol pyrophosphates, whereas all inositol pyrophosphates have the ability to bring about protein pyrophosphorylation, e.g. the yeast cyclin-CDK (cyclin-dependent kinase)–CDK inhibitor complex Pho80-Pho85-Pho81 interacts non-covalently with 1-IP_7_ but not with 5-IP_7_ [21], although both forms of IP_7_ and IP_8_ can pyrophosphorylate proteins in *S. cerevisiae* extracts [22].

In keeping with the role of inositol pyrophosphates as metabolic messengers, a few studies suggest that these molecules may participate in ribosome biogenesis [4,6]. Nucleolar proteins Nsr1/nucleolin, Srp40/Nopp140 and TCOF1 are substrates for IP_7_-mediated pyrophosphorylation [22,23]. The loss of Kcs1 partially suppresses the cold sensitivity observed in yeast cells carrying a mutant version of Rrs1, a protein involved in 60S ribosomal subunit assembly [24]. Inositol pyrophosphates control the activity of the histone deacetylase Rpd3L, which regulates transcriptional changes in response to stress, including genes involved in ribosome biogenesis [16]. In mammalian cells, IP_5_ 2-kinase, the enzyme that synthesizes IP_6_, has been shown to associate with the nucleolus, acting as a scaffold independent of its catalytic activity to promote rRNA synthesis [25].

We have now thoroughly examined the relationship between inositol pyrophosphates and ribosome synthesis. We note that *kcs1*Δ yeast cells display sensitivity to translation inhibitors, reduced protein synthesis and lower ribosome levels compared with wild-type (WT) cells. Our data reveal that RNA Pol I-mediated rRNA synthesis is substantially lowered in *kcs1*Δ yeast strain. We have identified three subunits of RNA Pol I–A190, A43 and A34.5–as being pyrophosphorylated by IP_7_, and note that transcription elongation by Pol I is compromised in *kcs1*Δ yeast cells.

## EXPERIMENTAL

All reagents, unless otherwise stated, were procured from Sigma-Aldrich, [^14^C]uracil from Ogene Systems, and [γ-^32^P]ATP, [^35^S]methionine/cysteine and [α-^32^P]UTP from JONAKI/BRIT.

### Strains, plasmids and growth conditions

The *S. cerevisiae* strains used in this study are listed in Supplementary Table S1. The DDY1810 *S. cerevisiae* strains came from Adolfo Saiardi [14], and the *kanMX4* cassette in *vip1*Δ and *kcs1*Δ*ddp1*Δ strains was removed by using the Cre-*loxP* recombination system [26]. The BY4741 *kcs1*Δ strain came from Beverley Wendland [27]. The NOY222 RPA190 shuffle strain carrying a complemented deletion of *RPA190*, and the plasmids encoding WT and serine-to-alanine mutant versions of RPA190 came from Herbert Tschochner [28]. An *rpa190* and *rpa34* double-mutant strain was generated by mating BY4741 *rpa34*Δ with NOY222 using standard yeast genetic techniques [29], and the resulting haploid strain was phenotyped for the presence of auxotrophy and drug resistance markers (see Supplementary Table S1). A genomic mutation on the *RPA43* gene was inserted into the NOY222 *rpa34*Δ strain and the BY4741 WT strain. The pRS314-*RPA43* (from Herbert Tschochner [30]) was used as a template for PCR-based site-directed mutagenesis to create a mutant version of *RPA43* (RPA43 S322/323/325A). Using homologous recombination methods, the indicated serine codons were substituted with alanine in the C-terminal tail of RPA43, by inserting the nourseothricin *N*-acetyltransferase (*nat1*) gene [31] between the 3′-UTR of *RPA43* and the 5′-UTR of the downstream gene, *UBC11* (see Supplementary Table S2). Plasmids encoding WT and mutant *RPA190* (RPA190 S1413/1415/1417A) were introduced into the indicated strains (see Supplementary Table S1) by shuffling, as described earlier [28]. Yeast expression plasmids are listed in Supplementary Table S3. WT and deletion mutants of *S. cerevisiae* were grown in YPD (yeast extract/peptone/dextrose; Difco) at 30°C. Yeast cells carrying expression plasmids were grown in synthetic complete (SC) medium without uracil (SC−Ura). Unless mentioned otherwise, experiments were carried out using the BY4741 strain.

### Drug sensitivity assay

Analysis of sensitivity to translation inhibitors was conducted in the DDY1810 *S. cerevisiae* strain background, which does not contain the kanr selection marker (see Supplementary Table S1). Sensitivity to 6-azauracil (6AU) was monitored in the BY4741 or NOY222 strain backgrounds (see Supplementary Table S1). As uracil is a competitive inhibitor of 6AU, the plasmid p416GPD, carrying the *URA3* gene [32] was introduced into BY4741-derived strains to complement the *URA3* deletion in this strain. Overnight cultures grown in YPD or SC−Ura, were diluted to an absorbance at 600 nm (*A*_600_) of 0.25, followed by 5-fold serial dilutions, and 3 μl of each dilution was spotted on a YPD agar plate containing the indicated concentrations of translation inhibitors, or an SC−Ura agar plate, containing the indicated concentrations of 6AU. Growth was monitored at 30°C for 2–3 days.

### Protein synthesis assay

Cells (1 *A*_600_ unit) from mid-log phase yeast cultures grown in YPD were labelled in SC medium without methionine (SC−Met) containing 25 μCi/ml of [^35^S]methionine/cysteine for 5 min. Cells were lysed by bead beating in TBS (20 mM Tris/HCl, pH 7.2, 0.9% NaCl) with a protease inhibitor cocktail, and centrifuged at 12000 ***g*** for 15 min at 4°C. Sodium deoxycholate (final concentration 0.1 mg/ml) was added to the supernatant and incubated on ice for 30 min. Trichloroacetic acid was added to a final concentration of 6%, followed by incubation on ice for 1 h and centrifugation at 15000 ***g*** for 15 min at 4°C. The pellet obtained was suspended in TBS and counted in a liquid scintillation counter (PerkinElmer Tri-carb 2900). The values obtained in counts per minute were plotted using GraphPad Prism (Graphpad Software, Inc.).

### Doubling time and viability assessment

Yeast grown overnight was subcultured in SC medium or YPD at *A*_600_ 0.1. Growth was monitored for 72 h by measuring the *A*_600_ of the culture at regular intervals, and the doubling time was calculated from the exponential phase of growth by linear regression analysis on a semi-logarithmic scale, using GraphPad Prism. To determine yeast cell mass, cells equivalent to 5 *A*_600_ units were harvested from mid-log and stationary phase cultures, and washed with PBS. Cell pellets were dried at 50°C for 20 min and the dry weight of yeast measured. To assess the cell number, cells in mid-log or stationary phase were counted using a Neubauer chamber (marienfeld-superior) or haemocytometer and the number of cells present in 1 *A*_600_ unit was calculated. Cell death was monitored by incubating yeast cells in 0.2% Trypan Blue solution (Sigma-Aldrich) for 10 min, and scoring dead cells that take up the dye. To monitor cell viability, cells equivalent to 10^−5^ *A*_600_ units from mid-log and stationary phase cultures were plated on YPD/agar, incubated at 30°C for 48 h, and colonies were counted to extrapolate viable cell count per *A*_600_ unit.

### Ribosome profiles

Ribosome profiles were generated as described earlier [33] with some modifications. Yeast cells were grown in YPD until the mid-log phase and treated with cycloheximide (50 μg/ml), chilled on an ice-salt bath for 2–5 min and centrifuged immediately at 4000 ***g***. Cells were lysed in 1 ml of lysis buffer [10 mM Tris, pH 7.4, 100 mM NaCl, 30 mM MgCl_2_, 50 μg/ml cycloheximide, 200 μg/ml heparin, in 0.2% diethyl pyrocarbonate (DEPC)-treated water] and centrifuged at 10000 ***g*** for 10 min at 4°C. Cell lysates equivalent to 10 *A*_254_ units were loaded on top of a 10–50% sucrose continuous gradient in buffer (50 mM Tris/HCl, pH 7.4, 50 mM NH_4_Cl, 12 mM MgCl_2,_ 1 mM DTT, 0.1% DEPC) and centrifuged at 100000 ***g*** for 6 h and 4°C in an SW41 rotor (Beckman). Ribosome levels were measured by gradient analysis on an Isco UV-6 gradient collector by monitoring the absorbance at 254 nm. To analyse individual ribosome subunits, lysates were resolved on a 10–30% sucrose continuous gradient in buffer lacking MgCl_2_.

### RNA extraction and analysis

Total RNA was isolated by hot phenol extraction as described earlier [34] with slight modifications. Cells (1 *A*_600_ unit) from mid-log phase yeast cultures grown in YPD were lysed in AE solution (50 mM sodium acetate, pH 5.3, 10 mM EDTA), containing 1% SDS and an equal volume of acid-buffered phenol, pH 4.3, followed by incubation at 65°C for 15 min with continuous shaking. Lysates were chilled on ice and centrifuged at 12000 ***g*** for 10 min. The aqueous phase was transferred to a tube containing an equal volume of chloroform, mixed well and centrifuged at high speed. RNA was precipitated by the addition of 50 μl of 3 M sodium acetate followed by 100% ethanol, and dissolved in DEPC-treated water. RNA was estimated by measuring the *A*_260_ using a spectrophotometer (Thermo Scientific ND-1000). To monitor rRNA levels, 10 μg of total RNA from each strain was resolved on a 1.2% formaldehyde/agarose gel.

RNA-labelling experiments were performed by harvesting mid-log phase yeast cells grown in YPD. Cells equivalent to 1 *A*_600_ unit were incubated in SC−Ura medium containing 3 μCi/ml of [^14^C]uracil for different lengths of time, and RNA was extracted as described previously. Equal amounts of total RNA were resolved on a formaldehyde agarose gel, stained with ethidium bromide and transferred to an N^+^ Hybond membrane (GE Life Sciences). Radiolabelled rRNA was detected using a phosphorimager scanner (Fujifilm FLA-9000, FUJIFILM Corp.). Pulse-chase analysis of rRNA was performed as described earlier [33], with slight changes. Yeast cells were harvested at an *A*_600_ of 0.5–0.7. The cells were washed and labelled in 1 ml of SC−Ura medium containing 3 μCi/ml of [^14^C]uracil for 5 min at 30°C. A chase was performed with SC medium containing 240 mg/l of unlabelled uracil. Samples were harvested 0, 1, 5, 15 and 20 min after the chase, and centrifuged at 12000 ***g*** for 1 min at 4°C. RNA was extracted, and incorporation of radioactivity was detected as described earlier.

### Protein purification and IP_7_ pyrophosphorylation reaction

Proteins were purified from the DDY1810 *S. cerevisiae* strain containing plasmids encoding GST-tagged proteins (see Supplementary Table S3), as described earlier [35]. The pyrophosphorylation reaction was performed with proteins bound to glutathione beads in the presence of IP_7_ reaction buffer (25 mM Hepes, pH 7.4, 50 mM NaCl, 6 mM MgCl_2_, 1 mM DTT) and 1 μCi of 5[β-^32^P]IP_7_ (120 Ci/mmol) at 37°C for 15 min. To the reaction mixture, LDS sample buffer (Invitrogen) was added and incubated at 95°C for 5 min. Proteins were resolved on a 4–12% gradient gel (Invitrogen) by NuPAGE (Life Technologies) and transferred to a PVDF membrane (GE Life Sciences). Radiolabelled proteins were detected using a phosphorimager (Fujifilm FLA-9000) and immunoblotted with an anti-GST antibody (Abcam).

### Chromatin immunoprecipitation

The chromatin immunoprecipitation assay was performed as described earlier [36] with slight modifications. Mid-log phase yeast cultures (45 ml) grown in YPD were subjected to cross-linking with 1% formaldehyde for 15 min at room temperature. Cross-linking was quenched by adding glycine to a final concentration of 0.1 M. Cells were washed in ice-cold TBS and lysed in 500 μl of ice-cold lysis buffer (50 mM Hepes, pH 7.5, 140 mM NaCl, 1% Triton X-100, 0.1% sodium deoxycholate, 1 mM EDTA, protease inhibitor cocktail) by bead beating. Chromatin was fragmented using a bath sonicator (Diagenode Diagnostics). Cell lysates were centrifuged at high speed and the supernatant was pre-cleared with normal rabbit IgG followed by Protein A beads (GE Life Sciences). Supernatant was collected and 10 μl of this lysate was taken as input. Immunoprecipitation of chromatin was performed by incubating the lysate with anti-GST antibody overnight at 4°C, followed by Protein A beads for 4 h. Beads were washed twice each in wash buffer I (50 mM Hepes, pH 7.5, 500 mM NaCl, 1% Triton X-100, 0.1% sodium deoxycholate, 1 mM EDTA, protease inhibitor cocktail), wash buffer II (10 mM Tris/HCl, pH 8.0, 1 mM EDTA, 250 mM LiCl, 0.75% NP-40, 0.75% sodium deoxycholate) and TE (10 mM Tris/HCl, pH 8.0, 1 mM EDTA) buffer. Chromatin was eluted in 100 μl of elution buffer (50 mM Tris/HCl, pH 8.0, 10 mM EDTA, 1% SDS) and incubated at 65°C overnight to reverse the cross-linking. DNA was extracted using a PCR purification kit (Qiagen). PCR reactions were set up with primers 5′-GCTAAGATTTTTGGAGAATAGC-3′ and 5′-GCCTACTCGAATTCGTTTCC-3′ to amplify the rDNA promoter, and primers 5′-TCAAACGGTGGAGAGAGTCG-3′ and 5′-ACCAATGGAATCGCAAGATGC-3′ to amplify the 5′-external transcribed spacer (5′-ETS). Real-time PCR was performed using Mesa Green 2X PCR MasterMix (Eurogentec) in a 20-μl reaction volume using 1 μl from the input sample and 3 μl from the immunoprecipitated sample (Applied Biosystems). Ct values of the immunoprecipitated samples were normalized to the adjusted Ct values of input, and data were plotted using GraphPad Prism.

### Transcription run-on analysis

The assay was performed as described in Elion and Warner [37]. Briefly, yeast cells equivalent to 1 *A_6_*_00_ unit were harvested from mid-log phase grown cultures, permeabilized with 0.5% sodium lauroyl-sarcosinate (sarkosyl), and incubated with 100 μl of reaction mix containing 100 μCi [α-^32^P]UTP (3000 Ci/mmol) for 10 min at 25°C. RNA was extracted and hybridized to plasmids blotted on Hybond N^+^ membranes in replicates, containing probes corresponding to different regions on the rDNA gene. These include the rDNA start (+1 to +177), 5′-ETS (+351 to +610), end-5′-ETS (+611 to +952) and non-transcribed spacer (NTS)-2. Empty TOPO plasmid (Life Technologies) and genomic DNA extracted from WT yeast cells were used as controls. Radioactivity was measured with phosphorimager scanning. Data were analysed by densitometry using AlphaEaseFC 4.0 and graphs were plotted using GraphPad Prism.

## RESULTS

### Inositol pyrophosphate-deficient yeast show reduced protein synthesis

**Figure 1:**
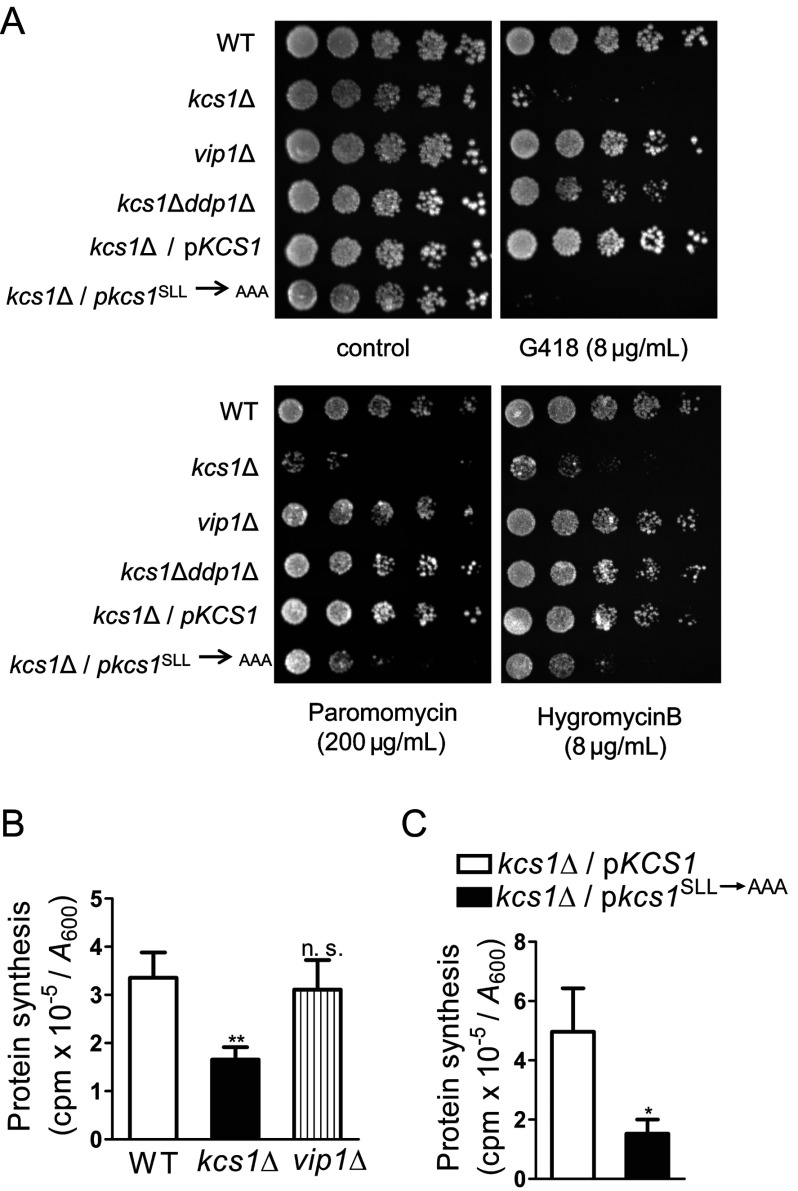
*S. cerevisiae* lacking Kcs1 displays a defect in translation (**A**) 5-fold serial dilutions of the indicated *S. cerevisiae* strains were plated on YPD with or without protein synthesis inhibitors, and incubated for 2–3 days at 30°C. Data represent three independent experiments. (**B**) Protein synthesis was measured in the indicated *S. cerevisiae* strains by pulse-labelling cells for 5 min with [^35^S]methionine/cysteine. Radioactivity incorporated into total protein, expressed as counts per min (cpm), was normalized to the absorbance (*A*_600_) of the labelled culture. Data are means±S.E.M. (*n*=4). (**C**) Protein synthesis was measured in *kcs1*Δ cells expressing either native or catalytically inactive forms of Kcs1, as described in (**B**). Data are means±S.E.M. (*n*=6). *P* values are from a two-tailed paired *t*-test (*P≤0.05; **P≤0.01; n.s. not significant, *P*>0.05).

We monitored ribosome function in yeast cells by growing them in the presence of aminoglycoside antibiotics, G418, paromomycin or hygromycin B, which disrupt the elongation cycle during polypeptide synthesis. We observed that *kcs1*Δ yeast cells, which have no detectable inositol pyrophosphates, are sensitive to low doses of these drugs; at these doses WT yeast cells show either no change in growth or mild sensitivity (Figure 1A). Conversely, *vip1*Δ yeast cells, which have higher levels of 5-IP_7_ than WT yeast cells but no 1-IP_7_, show no loss of viability at low doses of these inhibitors. We also tested the double knockout *kcs1*Δ*ddp1*Δ strain, which accumulates 1-IP_7_ but has no 5-IP_7_ or IP_8_. The additional removal of Ddp1 partially rescues the sensitivity of yeast cells lacking Kcs1 to G418, and completely eliminates sensitivity to paromomycin and hygromycin B. This suggests that the presence of either 1-IP_7_ or 5-IP_7_ is sufficient to confer resistance to translation inhibitors. The sensitivity of *kcs1*Δ yeast strain to translation inhibitors could reflect a defect in protein synthesis. Incorporation of radiolabelled methionine/cysteine into proteins is significantly reduced in *kcs1*Δ yeast cells compared with WT cells (Figure 1B), but, as expected, *vip1*Δ yeast cells show no change in the rate of protein synthesis.

To determine whether Kcs1 plays a role in maintaining protein synthesis levels as a function of its IP_6_ kinase activity, we reconstituted the *kcs1*Δ yeast strain with either active or catalytically inactive forms of Kcs1 [13]. Expression of active Kcs1 was able to rescue sensitivity to low doses of translation inhibitors (Figure 1A), and bring the rate of protein synthesis back to WT levels (Figure 1C). However, inactive Kcs1 was unable to rescue these defects (Figures 1A and 1C), suggesting that the presence of inositol pyrophosphates is essential to maintain optimum protein synthesis in yeast cells.

A lower rate of protein synthesis may be the underlying basis for the growth defect documented in *kcs1*Δ yeast strain [13,15]. We measured the growth rates of the yeast strains used in the present study. Although *kcs1*Δ yeast cells display a significant increase in doubling time when cultured in minimal medium, the defect is less pronounced in rich YPD medium (see Supplementary Table S4). The growth defect is not apparent when *kcs1*Δ cells are grown for 2 days on a YPD plate (Figure 1A), but is clearly visible on a minimal medium plate (results not shown), as observed by Dubois et al [13]. Expression of active, but not inactive, Kcs1 can rescue this growth defect (see Supplementary Table S4). As anticipated, *vip1*Δ yeast cells show no growth defect, and display a doubling time comparable with the WT strain. We also noted that, despite a decrease in growth rate, cell mass, cell number and viability in a rich medium are unaltered in *kcs1*Δ yeast strain (see Supplementary Figure S1).

### Reduced ribosome content and lower rRNA transcription in *kcs1*Δ yeasts

**Figure 2:**
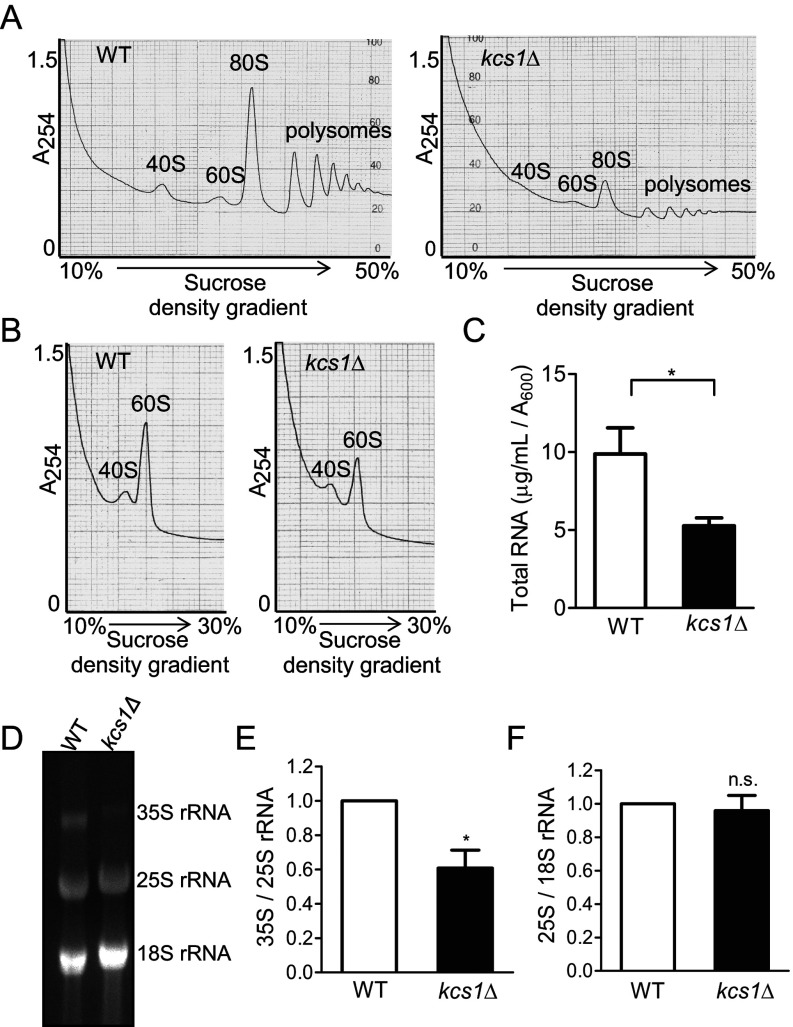
Ribosome content is reduced in yeast cells lacking IP_7_ (**A**) Polysomal profiles of equal concentrations of WT and *kcs1*Δ yeast lysates, as measured by absorbance at 254 nm (*A*_254_). The positions of 40S and 60S ribosomal subunits, 80S monosomes and polysomes are indicated. (**B**) Ribosomal subunit profiles of equal concentrations of WT and *kcs1*Δ yeast lysates. The positions of 40S and 60S subunits are indicated. Data represent two independent experiments. (**C**) Total RNA isolated from yeast cells, quantified by measuring *A*_260_, was normalized to the absorbance (*A*_600_) of the culture. Data are means±S.E.M. (*n*=6). (D–F) Total RNA (10 μg) isolated from yeast cells was resolved using denaturing agarose gel electrophoresis. (**D**) 35S, 25S and 18S rRNA were quantified by densitometry analysis. (**E**) Levels of 35S rRNA were compared with 25S rRNA and (**F**) levels of 25S rRNA with 18S rRNA; these ratios in *kcs1*Δ cells were normalized to WT cells. Data are means±S.E.M. (*n*=4). *P* values are from (**C**) a two-tailed paired *t*-test or (**E**, **F**) a one-sample *t*-test (*P≤0.05; n.s. not significant, *P*>0.05).

The lower rate of protein synthesis observed in *kcs1*Δ cells may be due to reduced ribosome levels or defects in ribosome activity. We therefore conducted a polysome analysis in WT and *kcs1*Δ yeast strains to determine the levels of monosomes and polysomes assembled on mRNA. The *kcs1*Δ cells display a significant reduction in monosome and polysome content (Figure 2A). Our findings are similar to those of Mizuta and colleagues, who observed that *kcs1*Δ yeast cells display reduced polysome levels when grown at 16°C [24], but, in contrast with their observation, we also note a decrease in 80S monosome levels in cells grown at 30°C. The decrease in polysomes could be due to a defect in the assembly of ribosomal subunits on mRNA, or reflect a decrease in total ribosome content. Under conditions in which individual ribosome subunits are detected, we noted a decrease in the levels of 40S and 60S subunits in *kcs1*Δ cells (Figure 2B). In a eukaryotic cell, rRNA makes up 60% of the total RNA [1,38]. Lower ribosome levels in *kcs1*Δ cells were therefore reflected in a 40% decrease in total RNA content (Figure 2C). However, when equal total RNA was visualized, we observed reduced levels of 35S pre-rRNA relative to mature 25S or 18S rRNA in *kcs1*Δ cells (Figures 2D and 2E). There was no difference in the ratio of 25S to 18S rRNA (Figure 2F), indicating that a defect in rRNA processing is unlikely to be the basis of lower ribosome levels.

**Figure 3:**
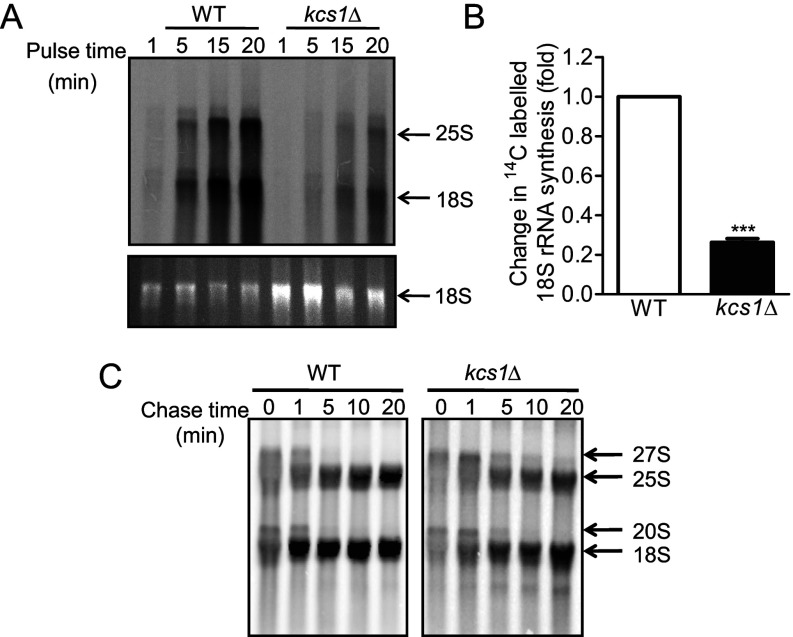
Synthesis of rRNA is reduced in yeast cells lacking IP_7_ (**A**) Yeast cells were labelled with [^14^C]uracil for the time indicated; RNA was isolated and resolved using denaturing agarose gel electrophoresis, detected by staining with ethidium bromide (lower panel) and transferred to a nylon membrane. Incorporation of radiolabelled uracil into rRNA was detected by phosphorimager scanning (upper panel). Data represent two independent experiments. (**B**) For the experiment described in (**A**), [^14^C]uracil incorporation into 18S rRNA (intensity of phosphorimager signal) was normalized to total 18S rRNA (intensity of ethidium bromide staining) at 5, 15 and 20 min, and these ratios in *kcs1*Δ cells were compared with those in WT cells. Data are means±S.E.M. (*n*=6). *P* values are from a one-sample *t*-test (***P≤0.001). (**C**) Yeast cells were labelled with [^14^C]uracil for 5 min and chased with excess unlabelled uracil for the time indicated. RNA was isolated, equal amounts of total RNA were resolved using denaturing agarose gel electrophoresis and the radioactivity was detected with phosphorimager scanning. The *kcs1*Δ blot, which had a fainter signal compared with the WT blot, was subjected to linear contrast adjustment to visualize bands. Data represent two independent experiments.

Reduced 35S rRNA levels may result from a reduction in rRNA synthesis by RNA Pol I. We examined rRNA synthesis by labelling cells for different lengths of time with [^14^C]uracil. The *kcs1*Δ cells display a substantial reduction in the incorporation of radiolabelled uracil into rRNA when normalized for total rRNA (Figures 3A and 3B). To track pre-rRNA processing, we pulse labelled cells with [^14^C]uracil for 5 min, and monitored radiolabelled rRNA levels during a chase with unlabelled uracil for different lengths of time. There was no accumulation of 27S and 20S precursor rRNAs in *kcs1*Δ cells, and no apparent difference in the rates of pre-rRNA processing to form mature 25S and 18S rRNA (Figure 3C). These data suggest that a reduction in rRNA synthesis may be the basis for the sensitivity to aminoglycoside antibiotics, lower ribosome level and reduced growth rate observed in yeasts lacking inositol pyrophosphates.

### RNA Pol I undergoes IP_7_-mediated pyrophosphorylation

**Figure 4:**
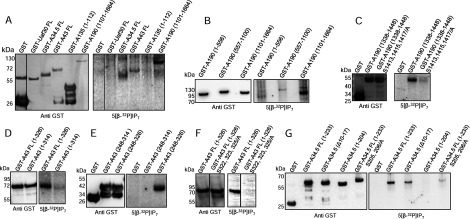
IP_7_ pyrophosphorylates RNA Pol I subunits (**A**) Purified, GST-tagged, full-length (FL) proteins Uaf30, A34.5, and A43, and the indicated fragments of A135 and A190, were incubated with 5[β-^32^P]IP_7_. Proteins were resolved using NuPAGE and transferred to a PVDF membrane. Pyrophosphorylation was detected by phosphorimager scanning (right) and proteins were detected by Western blotting (left). (**B**) Purified, GST-tagged, A190 fragments were pyrophosphorylated as in (**A**). (**C**) Purified GST-tagged A190 fragments corresponding to the native sequence and the indicated serine-to-alanine point mutants were pyrophosphorylated as in (A). (**D–F**) Pyrophosphorylation, as in (**A**), of purified, GST-tagged, FL fragments, and the indicated serine-to-alanine point mutants of A43. (**G**) Pyrophosphorylation, as in (**A**), of purified GST-tagged FL in-frame deletion, a C-terminally truncated fragment and the indicated serine-to-alanine point mutants of A34.5. To improve visualization, phosphorimager scans in (**A**), (**F**) and (**G**) were subjected to tonal range adjustment of the whole image using Adobe Photoshop (level adjustment). The start and end amino acid numbers of protein fragments are indicated in brackets. The dividing lines between lanes in panels (**A**) and (**F**) indicate the removal of non-essential lanes from a single original gel.

Sensitivity to translation inhibitors was observed in *kcs1*Δ strain, which displays no detectable inositol pyrophosphates, but not in *vip1*Δ or *kcs1*Δ*ddp1*Δ strains, which have only 5-IP_7_ or 1-IP_7_, respectively (see Figure 1A). Therefore, protein pyrophosphorylation, which can be effected by either form of IP_7_, may be the underlying molecular basis for the regulation of rDNA transcription by inositol pyrophosphates, and the target may be RNA Pol I and associated factors involved in rRNA synthesis. Regulation of Pol I activity during the logarithmic phase of growth occurs at the level of promoter binding [2] or transcription elongation [39]. Recruitment of RNA Pol I to the rDNA promoter involves four transcription factors, including the upstream activating factor (UAF) complex, TATA-binding protein (TBP), core factor (CF) complex and Rrn3 [40]. Transcription elongation is brought about by the 590-kDa 14-subunit RNA Pol I complex [1]. We examined databases curating mapped phosphorylation sites on these proteins, including PhosphoGRID (http://www.phosphogrid.org) and PhosphoPep (http://www.phosphopep.org), and picked those proteins that contain a pyrophosphorylation consensus site, i.e. two or more serine residues surrounded by acidic amino acids [17,22]. We narrowed the list down to five proteins containing an acidic serine motif: Uaf30, which is part of the UAF complex; and A190, A135, A43 and A34.5, which belong to the RNA Pol I elongation complex (see Supplementary Figure S2). We expressed these proteins in yeast as fusions to GST and examined their pyrophosphorylation with radiolabelled IP_7_. As A190 and A135 are large proteins of 186 kDa and 135 kDa, respectively, we tested only fragments of these proteins containing the predicted pyrophosphorylation sites. A43 and A34.5 are pyrophosphorylated by IP_7_, as is the C-terminal A190 fragment (Figure 4A). Uaf30 and an N-terminal fragment of A135, both of which contain potential pyrophosphorylation sites (see Supplementary Figures S2A and S2B), were found not to be IP_7_ substrates.

We went on to localize the site(s) of pyrophosphorylation on A190, A43 and A34.5. The A190 protein was divided into three non-overlapping fragments corresponding to the N-terminal, middle and C-terminal thirds of the protein. We detected robust pyrophosphorylation of the C-terminal fragment corresponding to residues 1101–1664, and weak pyrophosphorylation of the N-terminal and middle fragments (Figure 4B). The acidic serine motif within the C-terminal fragment maps to a Pol I-specific extended loop [41–43] which is absent from RNA polymerases II [42,44] and III [45]. A GST fusion of this region, corresponding to residues 1338–1448 (see Supplementary Figure S2C) was strongly pyrophosphorylated by IP_7_ (Figure 4C). Of the serine residues in this region, Ser^1413^, Ser^1415^ and Ser^1417^ have been shown to be phosphorylated [46], and are predicted to be sites for the protein kinase CK2 (phosphoGRID), which is known to prime serine residues for pyrophosphorylation by IP_7_ [22]. We replaced these three serine residues with alanine, and noted a dramatic reduction in the level of pyrophosphorylation (Figure 4C), although there was still some residual signal, suggesting that other serine residues in this fragment may be weak IP_7_ targets.

The A43 subunit has several mapped sites of serine phosphorylation (phosphoGRID; see Supplementary Figure S2D). In addition, we noted the presence of an acidic serine motif in the C-terminus of A43 (see Supplementary Figure S2D), which has not yet been identified as a phospho-site by mass spectrometry. Although full-length A43 was pyrophosphorylated by IP_7_, a version truncated at residue 314, lacking the C-terminal acidic serine motif, is not pyrophosphorylated (Figure 4D). We generated a fragment containing the C-terminal tail of A43 (residues 248–326), which is unique to Pol I and not present in its counterparts, Rpb7 [44] and C25 [45], in Pol II and Pol III, respectively. This fragment is robustly pyrophosphorylated by IP_7_, whereas truncation of the last 12 residues containing the acidic serine motif completely abolishes pyrophosphorylation (Figure 4E). Of the five serine residues located between 315 and 326 (see Supplementary Figure S2D), we replaced Ser^322^, Ser^323^ and Ser^325^ with alanine. Loss of these three serine residues led to the complete elimination of pyrophosphorylation on A43 (Figure 4F).

The Pol I-specific subunit, A34.5, has several acidic serine clusters (see Supplementary Figure S2E), of which Ser^10^, Ser^12^ and Ser^14^ have been identified as phosphoserines (phosphoGRID). However, removal of this motif by an in-frame deletion of residues 10–17 in full-length A34.5 had only a marginal effect on the extent of pyrophosphorylation (Figure 4G), suggesting that these are not the major targets of IP_7_. We therefore turned our attention to other acidic serine clusters in A34.5. We generated a construct of A34.5 corresponding to residues 1–204, which includes all acidic serine motifs except Ser^205^ and Ser^206^ in the C-terminal tail (see Supplementary Figure S2E). This fragment is not pyrophosphorylated by IP_7_ (Figure 4G). Interestingly, substitution of Ser^205^ and Ser^206^ with alanine in full-length A34.5 substantially reduced, but did not completely eliminate, IP_7_-mediated pyrophosphorylation (Figure 4G). It is possible that the highly charged C-terminal tail, which contains lysine/arginine residues in addition to aspartate/glutamate residues, assists in the binding of IP_7_ to A34.5, and that elimination of this tail completely abolishes pyrophosphorylation at weak sites. Our data therefore indicate that the main target in A34.5 for IP_7_-mediated pyrophosphorylation is the C-terminal tail (Ser^205^ and Ser^206^), and a weaker site is at the N-terminus (Ser^10^, Ser^12^ and Ser^14^).

### Elongation activity of Pol I is regulated by IP_7_

**Figure 5:**
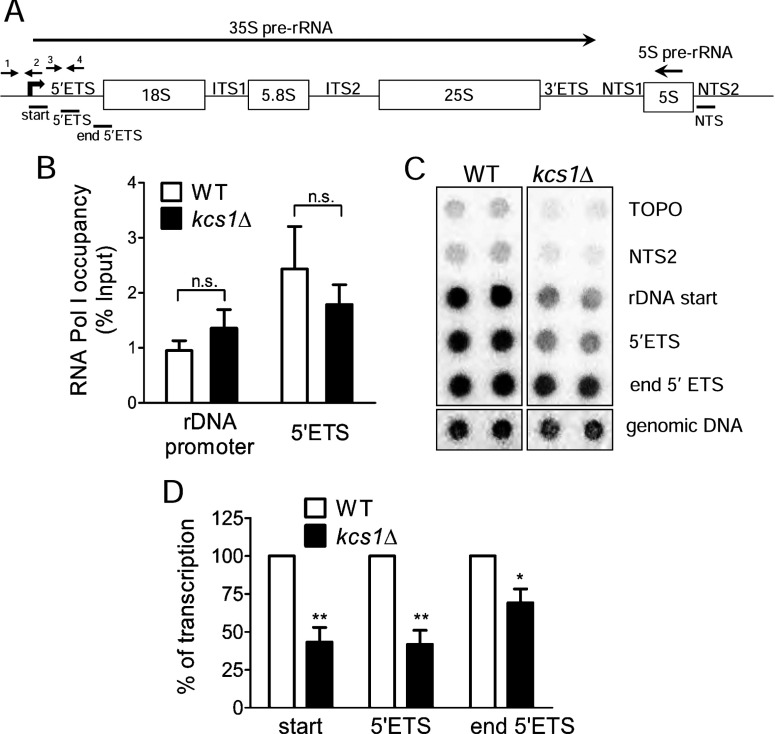
RNA Pol I elongation activity is lowered in *kcs1*Δ yeast strain (**A**) The 9.1-kb transcription unit of rDNA includes a 6.6-kb region encoding 35S pre-rRNA transcribed by RNA Pol I, a 121-bp region encoding 5S rRNA transcribed by RNA Pol III from the opposite strand and two NTSs. The 35S rDNA consists of 5′- and 3′-ETSs, two ITSs), and regions encoding the 18S, 5.8S and 25S mature rRNAs. Primers used for quantitative PCR (qPCR) are indicated by arrows. Primers 1 and 2 amplify the rDNA promoter (−174 to +57), and primers 3 and 4 amplify the 5′-ETS (+91 to +270). Probes used for transcription run-on analysis are indicated by solid lines. (**B**) Chromatin immunoprecipitation with GST-tagged A43, followed by qPCR with primers indicated in (**A**). Immunoprecipitated chromatin is expressed as a percentage of input chromatin in each sample. Data are means±S.E.M. (*n*=3). (**C**) Transcription run-on analysis using probes indicated in (A). The hybridization signals were quantified by densitometry analysis; the average intensity of the TOPO spots was considered as a background, and individual probe intensities were normalized to the genomic DNA signal. (**D**) These ratios in *kcs1*Δ cells were normalized to WT cells. Data are means±S.E.M. (*n*=4). *P* values are from (**B**) a two-tailed paired *t*-test or (**D**) a one-sample *t*-test (*P≤0.05; **P≤0.01; n.s. not significant, *P*>0.05).

The absence of IP_7_ may affect Pol I binding to the rDNA promoter, transcription initiation or elongation. *S. cerevisiae* has approximately 150 copies of tandem rDNA units arranged on chromosome XII, of which approximately half are transcriptionally active in exponentially growing cells [2]. Each rDNA unit encodes a 35S pre-rRNA transcribed by Pol I, which can be divided into regions coding for the mature 18S, 5.8S and 25S rRNA, two ETSs and two internal transcribed spacers (ITSs) which are cleaved during 35S pre-rRNA processing (Figure 5A). We used chromatin immunoprecipitation assays to monitor recruitment of the Pol I complex to the rDNA promoter. There is no difference in promoter binding by Pol I in WT and *kcs1*Δ cells (Figure 5B). To examine the levels of active elongating Pol I, we measured Pol I bound to the 5′-ETS, which occurs approximately 200 bp downstream of the promoter. There is no significant difference in Pol I occupancy of this region of the rDNA locus in WT and *kcs1*Δ yeast strains (Figure 5B). These results suggest that IP_7_ does not influence the recruitment of Pol I to the rDNA locus.

We then measured the elongation activity of Pol I in a nuclear run-on assay, which measures transcription by RNA polymerase that is already bound to DNA, while preventing recruitment of new polymerase molecules to DNA. As this assay utilizes permeabilized cells with an exogenous supply of nucleoside triphosphates (NTPs) for rRNA synthesis, it monitors the rate of transcription independent of the endogenous nucleotide pool, and is therefore not influenced by any effect of inositol pyrophosphates on cellular NTP levels. The levels of nascent transcript were significantly lower in *kcs1*Δ cells compared with WT cells (Figures 5C and 5D), indicating a reduction in the rate of transcription elongation by Pol I in the absence of inositol pyrophosphates. This finding agrees with the earlier data showing reduced levels of rRNA synthesis in *kcs1*Δ cells (see Figures 3A and 3B).

## DISCUSSION

The present study shows that inositol pyrophosphates participate in the regulation of ribosome synthesis in budding yeast. Yeast strains lacking the IP_6_ kinase Kcs1, which have no detectable IP_7_ and IP_8_, show sensitivity to translation inhibitors, whereas yeast cells that possess even one form of inositol pyrophosphate, i.e. 5-IP_7_ or 1-IP_7_, display a normal phenotype. The loss of yeast IP_6_ kinase affects the first step of ribosome synthesis, rRNA transcription by Pol I, leading to lower ribosome levels and reduced rates of protein synthesis. At the molecular level, inositol pyrophosphates can transfer their β-phosphate to phosphorylated serine residues on three Pol I subunits, A190, A43 and A34.5. Although inositol pyrophosphates do not appear to be necessary for loading Pol I on to the rDNA locus, they influence the rate of transcription elongation.

The pyrophosphorylated sequences that we have identified on A190, A34.5 and A43 fall within mobile regions, which are not visible in high-resolution crystal structures of the 14-subunit yeast RNA Pol I complex [42,43], but are contiguous with domains of known structure and function. Pyrophosphorylation on A190 occurs near a loop that sits in the DNA-binding cleft in inactive Pol I, but it is unclear how this region behaves in the elongating polymerase. The A34.5/A49 subcomplex is an intrinsic Pol I elongation factor [38,44], and the positively charged C-terminal tail of A34.5 anchors this subcomplex to the Pol I core [41–43]. Pyrophosphorylation of serine residues in this region could conceivably affect the attachment of the A34.5/A49 subcomplex on to the Pol I core, thereby affecting elongation activity. The A43/A14 heterodimer on the surface of Pol I [42–44] contributes to a ‘closed’ conformation of the Pol I clamp during elongation, supporting its high processivity [43]. The C-terminal tail of A43, which contains the pyrophospho-sites, invades the cleft of the neighbouring polymerase in an inactive dimer [42,43], but its role in the active elongating polymerase is unclear. The C-terminal region of A43 participates in the interaction of Pol I with the elongation factor Spt5 [47], which also interacts with A190 and A34.5 [38].

Pyrophosphorylation by IP_7_ occurs on pre-phosphorylated serine residues. Mass spectrometry analyses of the *S. cerevisiae* phosphoproteome (curated in PhosphoGRID) reveal that 12 of the 14 Pol I subunits are phosphorylated. A systematic analysis of 27 different phosphomutants in 5 Pol I core subunits, including A190, A43 and A34.5, revealed no significant growth phenotype and no effect on Pol I assembly or stability, suggesting that individual phosphorylated residues are non-essential [28]. However, treatment of Pol I with alkaline phosphatase lowers transcriptional activity *in vitro* [48], suggesting that concomitant phosphorylation at multiple sites is required for optimal Pol I activity. Our identification of A190 and A43 as IP_7_ substrates is consistent with an earlier report indicating that these Pol I subunits cannot be completely dephosphorylated on treatment with alkaline phosphatase [28], a property that is a hallmark of IP_7_-mediated pyrophosphorylation [22]. A global analysis of protein kinase interactions demonstrated that CK2 associates with Pol I in *S. cerevisiae* [49]. It is therefore possible that CK2 phosphorylates serine residues on Pol I, priming them for IP_7_-mediated pyrophosphorylation, as has been shown for other substrates of IP_7_ [17,22,50].

Pol I mutants defective in rRNA elongation display altered growth phenotypes in the presence of 6AU, which depletes the NTP pool [28,44,51]. As reported earlier [28,44], we noted a mild growth defect in the presence of 6AU in yeast cells lacking A34.5, and no change in growth or 6AU sensitivity in an A190 S1413/1415/1417/A mutant (see Supplementary Figure S3A). Substitution of alanine for Ser^322^, Ser^323^ and Ser^325^ in A43 did not lead to any significant change in growth or 6AU sensitivity (see Supplementary Figure S3A). However, when placed in the background of an A34.5 deletion, A43 S322/323/325A showed a substantial growth reduction in the presence of 6AU (see Supplementary Figure S3A), suggesting that pyrophosphorylation of A43 may indeed influence rRNA elongation. Interestingly, the significant reduction in protein synthesis observed in *kcs1*Δ cells (see Figure 1B) cannot be recapitulated by loss of the non-essential A34.5 subunit, removal of the pyrophosphorylated region in A43 and mutation of the major pyrophospho-site in A190 (see Supplementary Figure S3B). The influence of IP_7_ on transcription elongation may therefore result from a combined effect on the three Pol I pyrophosphorylated regions identified in the present study, and additional pyrophosphorylation events on other sites on Pol I or associated elongation factors. One such candidate is the phosphoprotein Nsr1, the *S. cerevisiae* homologue of nucleolin, a strong target of IP_7_-mediated pyrophosphorylation [23]. Mammalian nucleolin has been shown to stimulate Pol I elongation by aiding nucleosome displacement during transcription on chromatin templates, in addition to its role in pre-rRNA maturation and ribosome assembly [52]. Therefore, Nsr1 could potentially contribute to the role of IP_7_ in regulating transcription elongation by Pol I. However, unlike the *kcs1*Δ strain, deletion of Nsr1 results in defects in 35S pre-rRNA processing and an increase in the level of 60S subunits [33,53], suggesting that not all effects of IP_7_ on ribosome synthesis can be attributed to a change in Nsr1 function.

As the level of rDNA transcription by Pol I is a primary driver for cell proliferation [40], it is likely that the increased doubling time observed in *kcs1*Δ cells is due at least in part to reduced rRNA synthesis. Our finding also adds to the increasing repertoire of key metabolic processes regulated by inositol pyrophosphates. It has been argued that inositol pyrophosphates are metabolic messengers or energy sensors, rather than classic second messengers [4–6]. Inositol pyrophosphate levels are not rapidly altered by any particular stimulus, but instead have been shown to reflect intracellular ATP levels, most likely because the *K*_m_ value of IP_6_ kinases for ATP is approximately 1 mM, in the same range as the intracellular ATP concentration [4–6,54–56]. It was recently revealed that IP_6_ kinases can dephosphorylate IP_6_ to IP_5_ when there is a decrease in the cellular ATP:ADP ratio [56]. Cellular energy status has been shown to regulate Pol I-mediated rRNA synthesis, acting primarily through the TORC1 signalling pathway at the level of transcription initiation [57]. Although recent studies suggest that rDNA transcription is also regulated at the level of elongation [38,39], this has not been linked to the metabolic state of a cell. It is conceivable that IP_7_ acts as a conduit to transduce a decrease in intracellular ATP, signalling a reduction in RNA Pol I elongation activity and a lowering of ribosome biogenesis to conserve energy.

## Online data

Supplementary data
