# Supplementary material for: Inositol pyrophosphates regulate RNA polymerase I-mediated rRNA transcription in Saccharomyces cerevisiae
Source: Biochem J. 2015 Feb 6;466(Pt 1):105–14. doi: 10.1042/BJ20140798 (PMC4325516; doi:10.1042/BJ20140798)
Supplement: Supplementary data [file bj4660105ntsadd.pdf]

SUPPORTING INFORMATION

Supplementary Materials and Methods

All reagents, unless otherwise stated, were procured from Sigma-Aldrich, USA. [^{14}C]-labeled uracil was procured from Ogene Systems, Hyderabad, India. [γ-^{32}P]ATP, [^{35}S]Met/Cys, and [α-^{32}P]UTP were procured from JONAKI/BRIT, Hyderabad, India.

Yeast strains: All yeast strains used in this study are listed in Table S1. The DDY1810 *S. cerevisiae* strains were a gift from Dr. Adolfo Saiardi [1], and the *kanMX4* cassette in *vip1*Δ and *kcs1*Δ*ddp1*Δ strains was removed by using the Cre-*loxP* recombination system [2].The BY4741 *kcs1*Δ strain was a gift from Dr. Beverley Wendland [3]. The NOY222 RPA190 shuffle strain carrying a complemented deletion of *RPA190*, and the plasmids encoding WT and Ser to Ala mutant versions of RPA190 were gifted by Dr. Herbert Tschochner [4]. An *rpa190* and *rpa34* double mutant strain was generated by mating BY4741 *rpa34*Δ with NOY222 using standard yeast genetic techniques [5], and the resulting haploid strain was phenotyped for the presence of auxotrophy and drug resistance markers (Table S1). A genomic mutation on the *RPA43* gene was inserted into the NOY222 *rpa34*Δ strain and the BY4741 WT strain. pRS314-*RPA43* (a gift from Dr. Herbert Tschochner, [6]) was used as a template for PCR-based site-directed mutagenesis to create a mutant version of *RPA43* (RPA43$^{S322/323/325A}$). Using homologous recombination methods, the indicated Ser codons were substituted with Ala in the C-terminal tail of RPA43, by inserting the nourseothricin N-acetyltransferase (*nat1*) gene [7] between the 3'UTR of *RPA43* and 5'UTR of the downstream gene, *UBC11* (Table S4). Plasmids encoding WT and mutant *RPA190* (RPA190$^{S1413/1415/1417A}$) were introduced into the indicated strains (Table S1) by shuffling, as described earlier [4].

Drug sensitivity assay: Analysis of sensitivity to translations inhibitors was conducted in the DDY1810 *S. cerevisiae* strain background, that does not contain the kan^r selection marker (Table S1). Sensitivity to 6-azauracil (6AU) was monitored in the BY4741 or NOY222 strain backgrounds (Table S1). As uracil is a competitive inhibitor of 6AU, the plasmid p416GPD, carrying the *URA3* gene [8] was introduced into BY4741-derived strains to complement the *URA3* deletion in this strain. Overnight cultures grown in YPD or synthetic complete (SC) medium without uracil, were diluted to OD_{600} 0.25, followed by 5 fold serial dilutions, and 3µL of each dilution was spotted on a YPD-agar plate containing the indicated concentrations of translation inhibitors, or an SC-Ura agar plate, containing the indicated concentrations of 6AU. Growth was monitored at 30°C for 2-3 days.

Protein synthesis assay: 1 OD_{600} unit of cells from mid-log phase yeast cultures grown in YPD were labeled in SC medium without Methionine (SC-Met) containing 25 µCi/mL [^{35}S]Met/Cys for 5 min. Cells were lysed by bead-beating in Tris-buffered saline (20 mM Tris-HCl pH 7.2, 0.9% NaCl) with protease inhibitor cocktail and centrifuged at 12,000 ***g*** for 15 min at 4°C. Sodium deoxycholate (final concentration 0.1 mg/mL) was added to the supernatant and incubated on ice for 30 min. Trichloroacetic acid was added to a final

concentration of 6%, followed by incubation on ice for 1 h and centrifugation at 15,000 ***g*** for 15 min at 4°C. The pellet obtained was suspended in Tris-buffered saline and counted in a liquid scintillation counter (Perkin Elmer Tri-carb 2900). The cpm (counts per min) values obtained were plotted using GraphPad Prism.

Ribosome profiles: Ribosome profiles were generated as described earlier [9] with some modifications. Yeast were grown in YPD till mid-log phase and were treated with cycloheximide (50 µg/mL), chilled on an ice-salt bath for 2-5 min and centrifuged immediately at 4,000 ***g***. Cells were lysed in 1 mL lysis buffer (10 mM Tris pH 7.4, 100 mM NaCl, 30 mM $MgCl_2$, 50 µg/mL cycloheximide, 200 µg/mL heparin, in 0.2% diethyl pyrocarbonate (DEPC)-treated water) and centrifuged at 10,000 ***g*** for 10 min at 4°C. Cell lysates equivalent to 10 A_{254} units were loaded on top of a 10%-50% sucrose continuous gradient in buffer (50 mM Tris-HCl pH 7.4, 50 mM NH_4Cl, 12 mM $MgCl_2$, 1 mM DTT, 0.1% DEPC) and centrifuged at 100,000 ***g*** at 4°C for 6 h in an SW41 rotor (Beckman). Ribosome levels were measured by gradient analysis on an ISCO UV-6 gradient collector by monitoring absorbance at 254 nm. To analyse individual ribosome subunits, lysates were resolved on a 10%-30% sucrose continuous gradient in buffer lacking $MgCl_2$.

Doubling time and viability assessment: Overnight grown yeast were sub-cultured in SC medium or in YPD at 0.1 OD_{600}. Growth was monitored for 72 h by measuring OD_{600} of the culture at regular intervals, and doubling time was calculated from the exponential phase of growth by linear regression analysis on a semi-logarithmic scale, using GraphPad Prism.
To determine yeast cell mass, cells equivalent to 5 OD_{600} were harvested from mid-log and stationary phase cultures, and washed with PBS. Cell pellets were dried at 50°C for 20 min and the dry weight of yeast was measured. To assess the cell number, cells in mid-log or stationary phase were counted using a Neubauer chamber and the number of cells present in 1 OD_{600} was calculated. Cell death was monitored by incubating yeast cells in 0.2% trypan blue solution (Sigma-Aldrich) for 10 min, and scoring dead cells that take up the dye. To monitor cell viability, cells equivalent to 10^{-5} OD_{600} from mid-log and stationary phase cultures were plated on YPD-agar, incubated at 30°C for 48 h, and colonies were counted to extrapolate viable cell count per OD_{600}.

RNA extraction and analysis: Total RNA was isolated by hot phenol extraction as described earlier [10] with slight modifications. 1 OD_{600} unit of cells from mid-log phase yeast cultures grown in YPD were lysed in AE solution (50 mM CH_3COONa pH 5.3, 10 mM EDTA), containing 1% SDS and an equal volume of acid-buffered phenol, pH 4.3, followed by incubation at 65°C for 15 min with continuous shaking. Lysates were chilled on ice and centrifuged at 12,000 ***g*** for 10 min. The aqueous phase was transferred to a tube containing an equal volume of chloroform, mixed well and centrifuged at high speed. RNA was precipitated by the addition of 50 µL 3M sodium acetate followed by 100% ethanol, and dissolved in DEPC-treated water. RNA was estimated by measuring A_{260} using a spectrophotometer (Thermo Scientific ND-1000). To monitor rRNA levels, 10µg of total RNA from each strain was resolved on a 1.2% formaldehyde-agarose gel.

RNA labelling experiments were performed by harvesting mid-log phase yeast cells grown in YPD. Cells equivalent to 1 OD_{600} unit were incubated in SC-Ura medium containing 3 μCi/mL [^{14}C]uracil for different lengths of time, and RNA was extracted as described previously. Equal total RNA was resolved on a formaldehyde agarose gel, stained with ethidium bromide and transferred to an N^+ Hybond membrane (GE Life Sciences). Radiolabeled rRNA was detected using a phosphorimager scanner (Fujifilm FLA-9000). Pulse-chase analysis of rRNA was performed as described earlier [9], with slight changes. Yeast cells were harvested at an OD_{600} of 0.5-0.7. The cells were washed and labeled in 1 mL SC-Ura medium containing 3 μCi/mL [^{14}C]uracil for 5 min at 30°C. A chase was performed with SC medium containing 240 mg/L unlabeled uracil. Samples were harvested 0, 1, 5, 15 and 20 min after the chase, and centrifuged at 12,000 ***g*** for 1 min at 4°C. RNA was extracted, and incorporation of radioactivity was detected as described earlier.

ChIP: The assay was performed as described earlier [11] with slight modifications. 45 mL of mid-log phase yeast cultures grown in YPD were subjected to cross linking with 1% formaldehyde for 15 min at room temperature. Cross linking was quenched by adding glycine to a final concentration of 0.1 M. Cells were washed in ice cold Tris-buffered saline and were lysed in 500 μL of ice cold lysis buffer (50 mM HEPES pH 7.5, 140 mM NaCl, 1% Triton X-100, 0.1 % sodium deoxycholate, 1 mM EDTA**,** protease inhibitor cocktail) by bead beating. Chromatin was fragmented using a bath sonicator (Diagenode). Cell lysates were centrifuged at high speed and the supernatant was pre-cleared with normal rabbit IgG followed by Protein A beads (GE Life Sciences). Supernatant was collected and 10 μL of this lysate was taken as input. Immunoprecipitation of chromatin was performed by incubating the lysate with anti-GST antibody overnight at 4°C followed by Protein A beads for 4 h. Beads were washed twice each in wash buffer I (50 mM HEPES pH 7.5, 500 mM NaCl, 1% Triton X-100, 0.1 % sodium deoxycholate, 1 mM EDTA**,** protease inhibitor cocktail), wash buffer II (10 mM Tris-HCl pH 8.0, 1 mM EDTA, 250 mM LiCl, 0.75% NP-40, 0.75% sodium deoxycholate), and TE buffer. Chromatin was eluted in 100 μL of elution buffer (50 mM Tris-HCl pH 8.0, 10 mM EDTA, 1% SDS) and incubated at 65°C overnight to reverse the cross linking. DNA was extracted using a PCR purification kit (Qiagen). PCR reactions were set up with primers 5'GCTAAGATTTTTGGAGAATAGC3' and 5'GCCTACTCGAATTCGTTTCC3' to amplify the rDNA promoter, and primers 5'TCAAACGGTGGAGAGAGTCG3' and 5'ACCAATGGAATCGCAAGATGC3' to amplify the 5'ETS. Real-time PCR was performed using Mesa Green 2X PCR MasterMix (Eurogentec) in a 20 μL reaction volume using 1 μL from the input sample and 3 μL from the immunoprecipitated sample (Applied Biosystems). Ct values of the immunoprecipitated samples were normalised to the adjusted Ct values of input, and data were plotted using GraphPad Prism.

Table S1. *S. cerevisiae* strains used in this study and their corresponding genotypes.

**Strain**	**Genotype**	**Reference**
BY4741 WT	*MATa his3Δ1 leu2Δ0 met15Δ0 ura3Δ0*	[12]
kcs1Δ	BY4741 *kcs1Δ::kanMX4*	[3]
vip1Δ	BY4741 *vip1Δ::kanMX4*	[13]
kcs1Δ / p*KCS1*	BY4741 *kcs1Δ::kanMX4,* p*KCS1*	[14], this study
kcs1Δ / p*kcs1* $^{SLL \rightarrow AAA}$	BY4741 *kcs1Δ::kanMX4, pkcs1-S887A/L888A/L889A (URA3)*	[14], this study
rpa34Δ	BY4741 *rpa34Δ::kanMX4*	[13]
rpa43 $^{SSDS \rightarrow AADA}$	BY4741 *rpa43-S322A/S323A/S325A::nat1*	This study [3]
DDY1810 WT	*MATa leu2-3, 112 trp1-Δ901 ura3-52 prb1-1122 pep4-3 prc1-407*	[1]
kcs1Δ	DDY1810 *kcs1Δ::LEU2*	[1]
vip1Δ	DDY1810 *vip1Δ::loxP*	[1], this study
kcs1Δddp1Δ	DDY1810 *kcs1Δ::loxP, ddp1Δ::LEU2*	This study
kcs1Δ / p*KCS1*	DDY1810 *kcs1Δ:: LEU2, pKCS1*	[14], this study
kcs1Δ / p*kcs1* $^{SLL \rightarrow AAA}$	DDY1810 *kcs1Δ:: LEU2, pkcs1-S887A/L888A/L889A (URA3)*	[14], this study
NOY222	*MATα trp1-Δ1 his4-Δ401 leu2-3,112 ura3-52 can r rpa190Δ::URA3 pNOY20 (LEU2 CANs, RPA190)*	[15]
RPA190 WT	*MATα trp1-Δ1 his4-Δ401 leu2-3,112 ura3-52 can r rpa190Δ::URA3 pRS314-RPA190 (TRP1)*	[4]
rpa190 $^{SDSDS \rightarrow ADADA}$	*MATα trp1-Δ1 his4-Δ401 leu2-3,112 ura3-52 can r rpa190Δ::URA3 pRS314-rpa190-S1413/1415/1417A (TRP1)*	[4]
NOY222 *rpa34Δ*	*MAT*α leu2$^-$ ura3$^-$ *can r rpa190Δ::URA3 rpa34Δ::kanMX4 pNOY20 (LEU2 CANs, RPA190)*	This study
RPA190 WT<br>*rpa34Δ*	*MAT*α leu2$^-$ ura3$^-$ *can r rpa190Δ::URA3 rpa34Δ::kanMX4 pRS314-RPA190 (TRP1)*	This study
rpa190 $^{SDSDS \rightarrow ADADA}$<br>*rpa34Δ*	*MAT*α leu2$^-$ ura3$^-$ *can r rpa190Δ::URA3 rpa34Δ::kanMX4 pRS314-rpa190-S1413/1415/1417A (TRP1)*	This study
NOY222 *rpa34Δ*<br>*rpa43* $^{SSDS \rightarrow AADA}$	*MAT*α leu2$^-$ ura3$^-$ *can r rpa190Δ::URA3 rpa34Δ::kanMX4 rpa43-S322A/S323A/S325A::nat1 pNOY20 (LEU2 CANs, RPA190)*	This study
RPA190 WT<br>*rpa34Δ*<br>*rpa43* $^{SSDS \rightarrow AADA}$	*MAT*α leu2$^-$ ura3$^-$ *can r rpa190Δ::URA3 rpa34Δ::kanMX4 rpa43-S322A/S323A/S325A::nat1 pRS314-RPA190 (TRP1)*	This study
rpa190 $^{SDSDS \rightarrow ADADA}$<br>*rpa34Δ*<br>*rpa43* $^{SSDS \rightarrow AADA}$	*MAT*α leu2$^-$ ura3$^-$ *can r rpa190Δ::URA3 rpa34Δ::kanMX4 rpa43-S322A/S323A/S325A::nat1 pRS314-rpa190-S1413/1415/1417A (TRP1)*	This study

Table S2. Primers used in this study to clone RNA Pol I subunits in pYesGex6p2 yeast expression vector [16]. *Inserts generated by overlap-extension PCR.

**Plasmid**	**Primers used to amplify insert**
pYesGex6p2	Yeast expression vector [16]
Uaf30FL	Uaf30-F: CTGATGGATCCATGGCTGAATTAAACGATTATAGTAC<br>Uaf30-R: CATGAGCGGCCGCTCAAATGCCCTTTGTATCTGATTG
A135 (1-112)	A135-F: TGATCGGATCCATGAGCAAAGTGATTAAGCC<br>A135-112-R: CATGAGCGGCCGCATCACCATCGTTAGACATTG
A190 (1-556)	A190-F: CTGATGGATCCATGGATATTTCTAAACC<br>A190-556-R: CATGACTCGAGCTATGCCTTACGTTGCTCTAC
A190 (557-1100)	A190-557-F: CTGATGGATCCTTAGCTAACCAACTATTGACAC<br>A190-1100-R: CATGACTCGAGCTACTTTTTAGAGTACTTCAAAG
A190 (1101-1664)	A190-1101-F: CTGATGGATCCACTTTGAAATACAGAAAGAA<br>A190-R: CATGAGCGGCCGCCTAAGCCGCATTTGGAACC
A190 (1338-1448)	A190-1338-F: ATCTGGGATCCAGAACTACAGGACCAGACATTG<br>A190-1448-R: CTATGCTCGAGATTATTGTTAGCTTCCACAATACTC
*A190 (1338-1448)<br>S1413,1415,1417/A	A190-1338-F: ATCTGGGATCCAGAACTACAGGACCAGACATTG<br>A190-SA-R: CAGCGTCAGCATCAGCTTCTTTATCGGAATCGATACCTTCTTCGTCAGAAG<br>A190-SA-F: GATTCCGATAAAGAAGCTGATGCTGACGCTGAGGACGAAGACGTTGACATG<br>A190-1448-R: CTATGCTCGAGATTATTGTTAGCTTCCACAATACTC
A43FL (1-326)	A43-F: TGATCGGATCCATGTCACAAGTAAAAAGAGCC<br>A43-R: CATGAGCGGCCGCCTAATCACTATCACTCGATTC
A43 (1-314)	A43-F: TGATCGGATCCATGTCACAAGTAAAAAGAGCC<br>A43-314-R: TACTGCGGCCGCTAGGGTGTTTTCCTCGTATACGATC
A43FL S322,323,325/A	A43-F: TGATCGGATCCATGTCACAAGTAAAAAGAGCC<br>A43-SA-R: CATGACTCGAGCTAATCAGCATCAGCCGCTTCACCATCATTGCTTTCACTG
A34FL (1-233)	A34-F: TGATCGGATCCATGTCCAAGCTTTCGAAAG<br>A34-R: CATGAGCGGCCGCTCAATCTCTATGTTTCTTTTTC
A34 (Δ10-17a.a)	A34-del-F:<br>TGATCGGATCCATGTCAAAACTTTCTAAGGACTACGTCGACGATGAAGTGATATCAAACGAGTTCAGC<br>A34-R: CATGAGCGGCCGCTCAATCTCTATGTTTCTTTTTC
A34 (1-204)	A34-F: TGATCGGATCCATGTCCAAGCTTTCGAAAG<br>A34-204-R: CATGACTCGAGCTACTCCTCTTCATCGTCGTGATGTG
*A34FL (1-233)S205,206/A	A34-F: TGATCGGATCCATGTCCAAGCTTTCGAAAG<br>A34-SA-R: CCTTCTTCTTTTTCTTCTTTTCAGCAGCCTCCTCTTCATCGTCGTGATG<br>A34-SA-F:<br>CAAAGAAGCGTTCACATCACGACGATGAAGAGGAGGCTGCTGAAAAGAAGAAAAAGAAGAAGG<br>A34-R: CATGAGCGGCCGCTCAATCTCTATGTTTCTTTTTC

Table S3. Doubling time of yeast strains. Growth of the indicated yeast strains at 30°C in rich medium (YPD), synthetic complete (SC) medium or SC medium without uracil (SC-Ura) was monitored up to 48 h by measuring OD_{600} of the culture at regular intervals. The doubling time was calculated from the logarithmic phase of growth. Data are mean ± SEM (n=4).

S. cerevisiae (BY4741)	Growth condition	Doubling time (h)
WT	YPD	1.72 ± 0.08
*kcs1*Δ	YPD	2.15 ± 0.01
WT	SC	1.83 ± 0.03
*kcs1*Δ	SC	2.96 ± 0.16
*vip1*Δ	SC	2.0 ± 0.01
*kcs1*Δ / p*KCS1*	SC-Ura	1.9 ± 0.02
*kcs1*Δ / p*kcs1*$^{SLL\rightarrow AAA}$	SC-Ura	2.97 ± 0.20

Table S4. Primers used in this study to introduce site-specific genomic mutations in *rpa43* to replace codons encoding Ser 322, 323 and 325 with codons encoding Ala.

Fragment name	Primers used to amplify the fragment
A43 3′	A43-gen-F: AGATCGTATACGAGGAAAACACCAGTGAAAGCAATGATGGTGAAGCAGCTGATGCTGATTAGGACGGGAAAAATGAG<br>A43-gen-R: CACGGCGCGCCTAGCAGCGGAAGATTGTCGAATTTGCCACGGTTTAGC
NAT 5′	NAT-5-F: GCTAAACCGTGGCAAATTCGACAATCTTCCGCTGCTAGGCGCGCCGTG<br>NAT-5-R: TCTGTTCCAACCAGAATAAG
NAT 3′	NAT-3-F: GTCTACTACTTTGGATGATAC<br>NAT-3-R: GTCAGAAGCATGTTTTGTGTCGCGGCCGCTGACGAAGTTCCTATTC
Ubc11 5′	UBC11-F: GAATAGGAACTTCGTCAGCGGCCGCGACACAAAACATGCTTCTGAC<br>UBC11-R: ATTATCGTCTACGGGAAACGCAC

SUPPLEMENTARY REFRENCES

1 Onnebo, S. M. and Saiardi, A. (2009) Inositol pyrophosphates modulate hydrogen peroxide signalling. Biochem. J. **423**, 109-118

2 Gueldener, U., Heinisch, J., Koehler, G. J., Voss, D. and Hegemann, J. H. (2002) A second set of loxP marker cassettes for Cre-mediated multiple gene knockouts in budding yeast. Nucleic Acids Res. **30**, e23

3 Saiardi, A., Sciambi, C., McCaffery, J. M., Wendland, B. and Snyder, S. H. (2002) Inositol pyrophosphates regulate endocytic trafficking. Proc. Natl. Acad. Sci. U.S.A. **99**, 14206-14211

4 Gerber, J., Reiter, A., Steinbauer, R., Jakob, S., Kuhn, C. D., Cramer, P., Griesenbeck, J., Milkereit, P. and Tschochner, H. (2008) Site specific phosphorylation of yeast RNA polymerase I. Nucleic Acids Res. **36**, 793-802

5 Guthrie, C. and Fink, G. R. (1991) Guide to yeast genetics and molecular biology. Academic Press Inc., Harcourt, Brace, Jovanovich, San Diego, California, U.S.A.

6 Peyroche, G., Milkereit, P., Bischler, N., Tschochner, H., Schultz, P., Sentenac, A., Carles, C. and Riva, M. (2000) The recruitment of RNA polymerase I on rDNA is mediated by the interaction of the A43 subunit with Rrn3. EMBO J. **19**, 5473-5482

7 Goldstein, A. L. and McCusker, J. H. (1999) Three new dominant drug resistance cassettes for gene disruption in *Saccharomyces cerevisiae*. Yeast **15**, 1541-1553

8 Mumberg, D., Muller, R. and Funk, M. (1995) Yeast vectors for the controlled expression of heterologous proteins in different genetic backgrounds. Gene **156**, 119-122

9 Lee, W. C., Zabetakis, D. and Melese, T. (1992) NSR1 is required for pre-rRNA processing and for the proper maintenance of steady-state levels of ribosomal subunits. Mol. Cell. Biol. **12**, 3865-3871

10 Laferte, A., Favry, E., Sentenac, A., Riva, M., Carles, C. and Chedin, S. (2006) The transcriptional activity of RNA polymerase I is a key determinant for the level of all ribosome components. Genes Dev. **20**, 2030-2040

11 Blattner, C., Jennebach, S., Herzog, F., Mayer, A., Cheung, A. C., Witte, G., Lorenzen, K., Hopfner, K. P., Heck, A. J., Aebersold, R., et al. (2011) Molecular basis of Rrn3-regulated RNA polymerase I initiation and cell growth. Genes Dev. **25**, 2093-2105

12 Brachmann, C. B., Davies, A., Cost, G. J., Caputo, E., Li, J., Hieter, P. and Boeke, J. D. (1998) Designer deletion strains derived from *Saccharomyces cerevisiae* S288C: a useful set of strains and plasmids for PCR-mediated gene disruption and other applications. Yeast **14**, 115-132

13 Giaever, G., Chu, A. M., Ni, L., Connelly, C., Riles, L., Veronneau, S., Dow, S., Lucau-Danila, A., Anderson, K., Andre, B., et al. (2002) Functional profiling of the *Saccharomyces cerevisiae* genome. Nature **418**, 387-391

14 Dubois, E., Scherens, B., Vierendeels, F., Ho, M. M., Messenguy, F. and Shears, S. B. (2002) In *Saccharomyces cerevisiae*, the inositol polyphosphate kinase activity of Kcs1p is required for resistance to salt stress, cell wall integrity, and vacuolar morphogenesis. J. Biol. Chem. **277**, 23755-23763

15 Wittekind, M., Dodd, J., Vu, L., Kolb, J. M., Buhler, J. M., Sentenac, A. and Nomura, M. (1988) Isolation and characterization of temperature-sensitive mutations in RPA190, the gene encoding the largest subunit of RNA polymerase I from *Saccharomyces cerevisiae*. Mol. Cell. Biol. **8**, 3997-4008

16 Werner, J. K., Jr., Speed, T. and Bhandari, R. (2010) Protein pyrophosphorylation by diphosphoinositol pentakisphosphate (InsP7). Methods Mol. Biol. **645**, 87-102

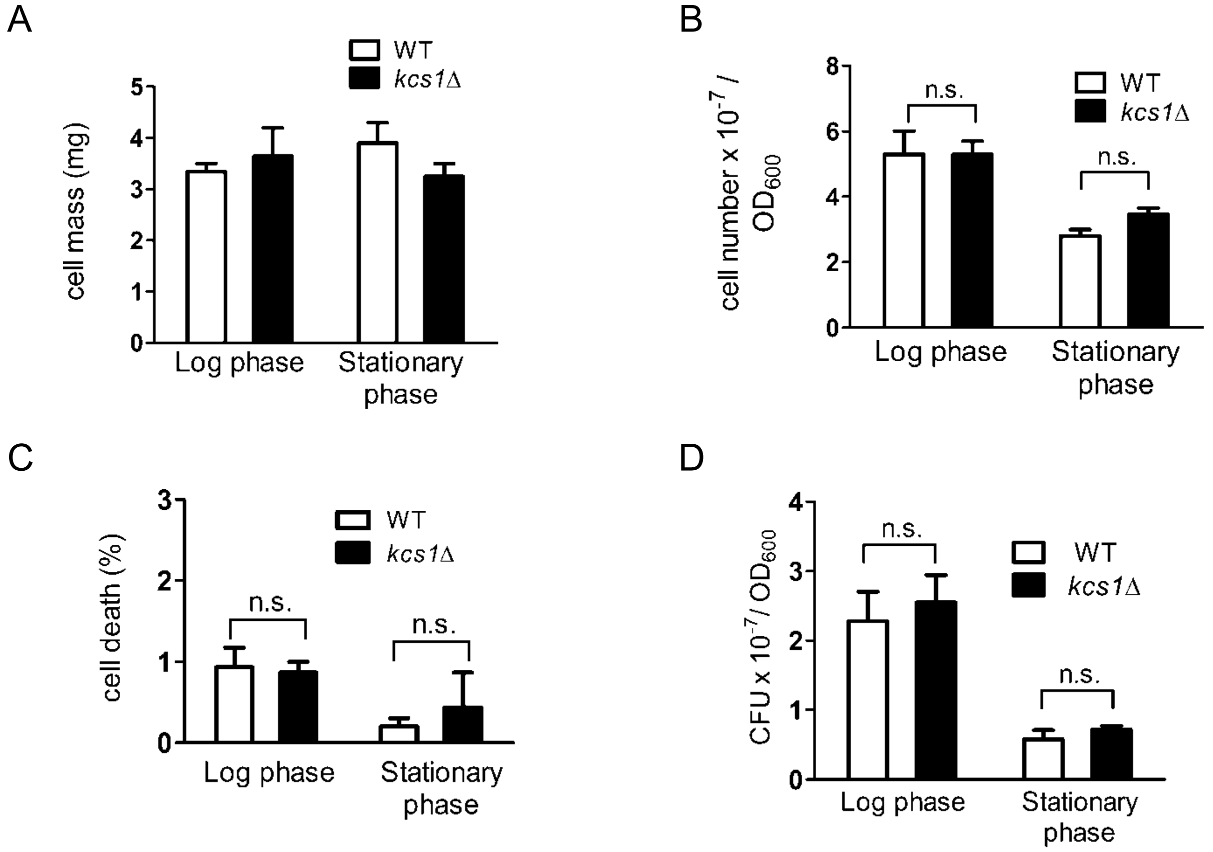

Figure S1. Viability and cell mass are unaltered in *kcs1*Δ yeast. (*A*) Yeast cells equivalent to 5 OD_{600} were harvested from log phase and stationary phase cultures and dry weight was measured. Data are mean ± range (n=2). (*B*) Cells from log phase and stationary phase cultures were counted using a Neubauer chamber and represented as total number of cells present in 1 OD_{600} of yeast culture. Data are mean ± SEM (n=3). (*C*) Cells from log phase and stationary phase cultures were stained with trypan blue. Dead cells were counted and represented as a percentage of total cell count. Data are mean ± SEM (n=3). (*D*) Cells from log phase and stationary phase cultures were plated on YPD-agar plates and colonies were counted to determine the number of viable cells per 1 OD_{600} unit (CFU colony forming units). Data are mean ± SEM (n=4). P values are from a 2-tailed paired *t* test (n.s. not significant)

A **Uaf30**

```
   1 MAELNDYSTM IDILLSDMDL ETVTTKKVRM ALKEVYAIDV ESQGKAINKL IRKHLDLVKE
  61 RPRFERSLED LLKENATLAI ELTKEITVSK RSSGEEKNDS ETKGTHVEKK KGTVSKSPIS
 181 TRKVTLSKSL ASLLGEHELT RTEVVRRLWA YIKAHNLQNP NNKKEILCDE KLELILGKST
 241 NMFEMHKILA SHMTEPKKIS DCPPLIQEVR RKEKPIVSDS EQSDTKGI
```

B **A135 fragment**

```
   1 MSKVIKPPGQ ARTADFRTLE RESRFINPPK DKSAFPLLQE AVQPHIGSFN ALTEGPDGGL
  61 LNLGVKDIGE KVIFDGKPLN SEDEISNSGY LGNKLSVSVE QVSIAKPMSN DG
```

C **A190 fragment**

```
1338                       RTT GPDIGVAVPR LQTDVANSSS NSKRLEEDND EEQSHKKTKQ
1381 AVSYDEPDED EIETMREAEK SSDEEGIDSD KESDSDSEDE DVDMNEQINK SIVEANNNMN
1441 KVQRDRQS
```

D **A43**

```
   1 MSQVKRANEN RETARFIKKH KKQVTNPIDE KNGTSNCIVR VPIALYVSLA PMYLENPLQG
  61 VMKQHLNPLV MKYNNKVGGV VLGYEGLKIL DADPLSKEDT SEKLIKITPD TPFGFTWCHV
 121 NLYVWQPQVG DVLEGYIFIQ SASHIGLLIH DAFNASIKKN NIPVDWTFVH NDVEEDADVI
 181 NTDENNGNNN NEDNKDSNGG SNSLGKFSFG NRSLGHWVDS NGEPIDGKLR FTVRNVHTTG
 241 RVVSVDGTLI SDADEEGNGY NSSRSQAESL PIVSNKKIVF DDEVSIENKE SHKELDLPEV
 301 KEDNGSEIVY EENTSESNDG ESSDSD
```

E **A34.5**

```
   1 MSKLSKDYVS DSDSDDEVIS NEFSIPDGFK KCKHLKNFPL NGDNKKKAKQ QQVWLIKFPS
  61 NVDISKLKSL PVDFESSTTM TIDKHDYKIM DDTDIESSLT QDNLSNMTLL VPSESKESLK
 121 IASTAKDNAP LQFDKVFSVS ETAKIPAIDY SKVRVPRKDV PKVEGLKLEH FATGYDAEDF
 181 HVAEEVKENK KEPKKRSHHD DEEESSEKKK KKKEKREKRE KKDKKDKKKK HRD
```

Figure S2. Amino acid sequences of RNA Pol I components tested for pyrophosphorylation by IP$_7$. (*A*) Sequence of full length Uaf30, a subunit of upstream activating factor (UAF). (*B*) Sequence of a fragment of A135 (amino acids 1-112), the second largest subunit of RNA Pol I. (*C*) Sequence of a fragment of A190 (amino acids 1338-1448) corresponding to the extended loop of RNA Pol I. (*D*) Sequence of full length A43. (*E*) Sequence of full length A34.5. All sequences are annotated as follows: previously determined phosphorylated serines (curated in PhoshoGRID) are underlined, serine residues within an acidic serine motif are coloured red, and pyrophosphorylated serine residues identified in this study are highlighted in yellow.

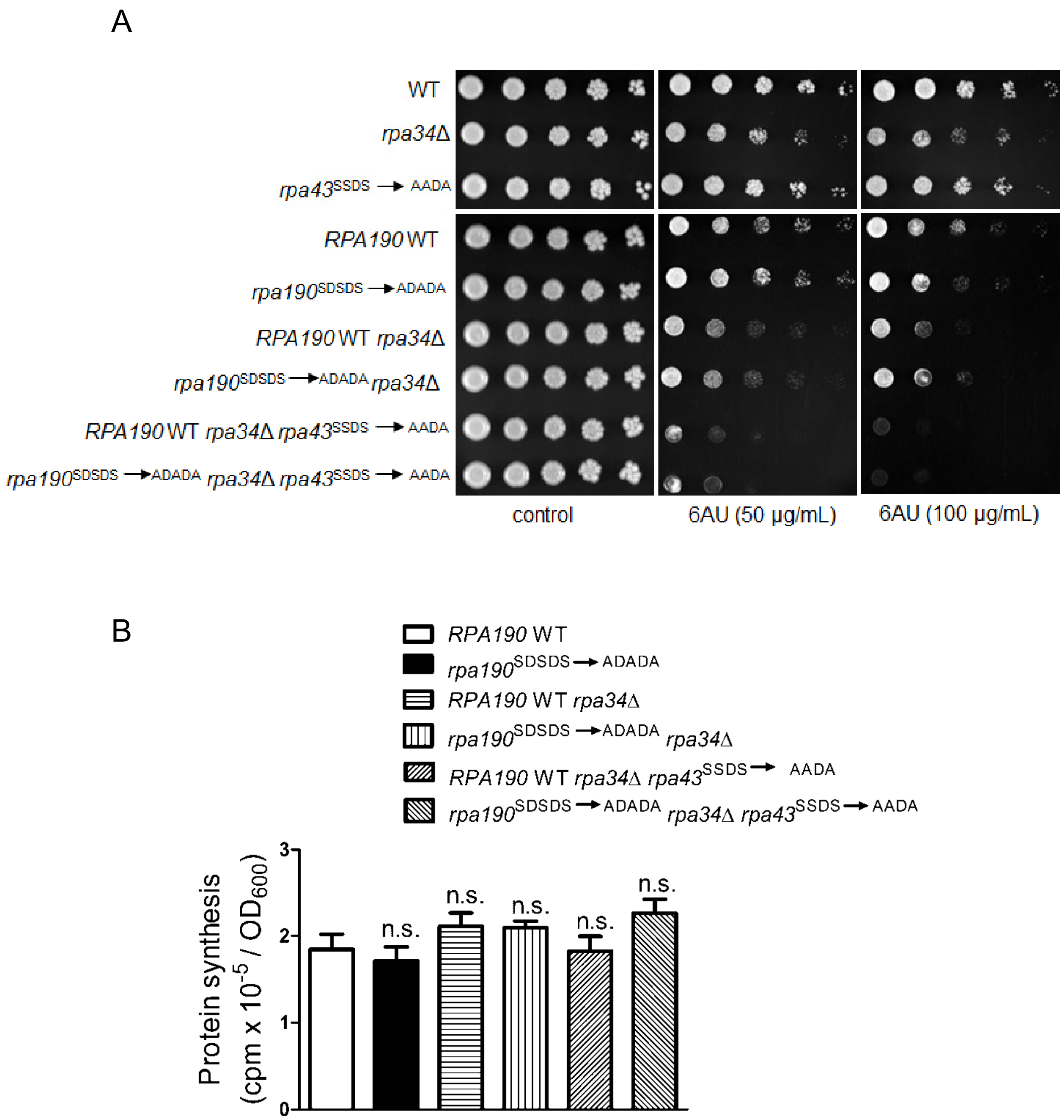

Figure S3. Phenotypic analysis of RNA Pol I mutants: (*A*) 5-fold serial dilutions of the indicated *S. cerevisiae* strains were plated on SC-Ura medium containing 6-azauracil (6AU), and incubated for 2-4 days at 30°C. (*B*) Protein synthesis was measured in the indicated *S. cerevisiae* strains by pulse-labeling cells for 5 min with [^{35}S]Met/Cys. Radioactivity incorporated into total protein expressed as counts per min (cpm) was normalised to the optical density (OD_{600}) of the labeled culture. Data are mean ± SEM (n=4). P values are from a 2-tailed paired *t* test (n.s. not significant, $P > 0.05$). See Supplementary Table S1 for a detailed description of the *S. cerevisiae* strains used in these analyses.
